# The Zen of XEN: insight into differentiation, metabolism and genomic integrity

**DOI:** 10.1038/s41419-018-1120-x

**Published:** 2018-10-22

**Authors:** Mohamed I. Gatie, Amy R. Assabgui, Gregory M. Kelly

**Affiliations:** 1 Molecular Genetics Unit, Department of Biology, The University of Western Ontario, London, ON Canada; 2 Department of Anatomy and Cell Biology, The University of Western Ontario, London, ON Canada; 3 Collaborative Graduate Specialization in Developmental Biology, The University of Western Ontario, London, ON Canada; 4 Department of Paediatrics, The University of Western Ontario, London, ON Canada; 5 Department of Physiology and Pharmacology, The University of Western Ontario, London, ON Canada; 6 Child Health Research Institute, London, ON Canada; 7 Ontario Institute for Regenerative Medicine, Toronto, ON Canada

## From fertilization to implantation

Fertilization of the mouse egg takes place in the oviduct, and following rounds of cell divisions the blastocyst forms and is comprised of three cell lineages. The epiblast houses naive or preimplantation embryonic stem cells (ESCs), which express OCT4, NANOG, REX1, and FGF4^1^. Once implanted into the uterine wall, naïve ESCs differentiate towards primed ESCs and continue to express OCT4 and NANOG, alongside FGF5 and T^1^. Collectively, cells of the epiblast give rise to the embryo proper. As for extraembryonic lineages, the trophectoderm, which gives rise to the placenta, is made up of trophoblast stem cells (TSCs)-expressing CDX2^1^. The third lineage consist of cells that form extraembryonic endoderm (XEN), which express GATA4, GATA6, SOX7, and SOX17^2^. XEN cells differentiate into parietal or visceral endoderm cells, and are essential for the survival and patterning of the embryo^2,3^. Despite the many studies that have focused on the derivation, maintenance, and differentiation of naïve and primed ESCs, fewer have addressed these in regard to extraembryonic lineages, specifically XEN cells.

## Mammalian embryos transition through distinct metabolic profiles

Our understanding of stem cells and their ability to self-renew and differentiate is corroborated by changes in global gene and protein expression, and the epigenetic modifications. Although these “–omic” approaches provide invaluable insight into the various characteristics that stem cells share, or what makes them unique from other cells, one common feature is their pluripotency, which is a topic of ongoing investigation. In the past decade, attention has shifted towards understanding the metabolic landscape of early mammalian embryos^4^. Glucose metabolism provides ATP for energy expenditure and substrates for anabolism that assists in modulating the epigenome. While most somatic cells use mitochondrial oxidative phosphorylation (OXPHOS) to generate ATP, Otto Warburg discovered that, despite having sufficient oxygen levels for OXPHOS metabolism, cancer cells rely on glycolysis to produce ATP^5^. This phenomenon, termed the Warburg effect, also occurs in stem cells^6^, where naïve ESCs utilize glycolysis and OXPHOS to generate ATP, while primed ESCs are exclusively glycolytic despite having structurally mature mitochondria^5^. Surprisingly, the appearance of these mitochondria in primed ESCs would suggest that they are capable of using OXPHOS, but detailed analysis has revealed that these cells express low levels of cytochrome *c* oxidase, thus reducing mitochondrial respiratory capacity^7^. As for extraembryonic lineages, we know TSCs use OXPHOS metabolism to generate ATP for energy^8^, and to power the Na^+^,K^+^–ATPase pump for blastocoel formation^9^, but we need a better understanding of the metabolic profile(s) in XEN cells to paint a complete picture of the events taking place in early development.

## The metabolic state of XEN cells

Our best understanding of the metabolic landscape of XEN cells comes from proteomic analysis^10^. Rate-limiting enzymes in glycolysis, including hexokinase 2 and glucose transporter 1, are downregulated during XEN induction; however, other enzymes remain unchanged or are elevated^10^. In fact, we have shown that lactate dehydrogenase A (LDHA), which catalyzes the conversion of pyruvate to lactate, is upregulated in embryo-derived XEN cells, while LDHB, which catalyzes the reverse reaction, is downregulated (unpublished data). Additionally, XEN induction is accompanied by an increase in the levels of enzymes involved in the TCA cycle and electron transport chain (ETC), yet mitochondrial biogenesis proteins are downregulated^10^. These seeming discrepancies suggest that the metabolome of XEN cells might be more complex than that of ESCs and TSCs, and thus further detailed interrogation is warranted.

## Factors influencing metabolism, differentiation, and stem cell quality

**Fig. 1:**
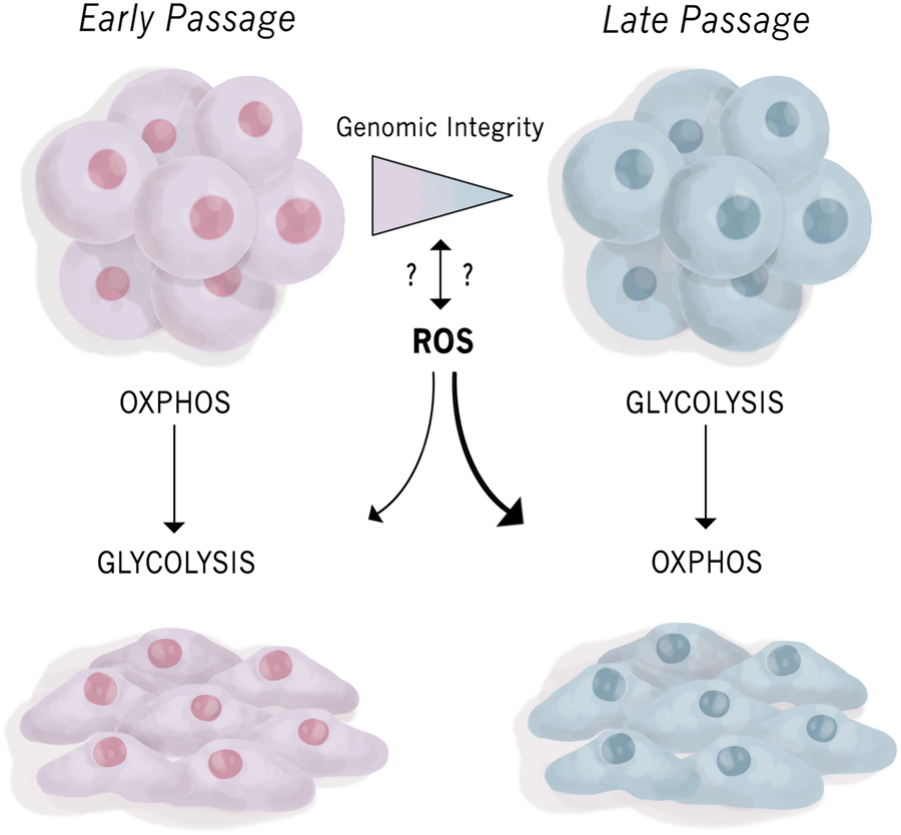
Differentiation potential, metabolic profile, and genomic integrity of early- versus late-passage F9 cells. F9 embryonal carcinoma stem-like cells differentiate towards a XEN lineage and switch their metabolic profile depending on their passage. Early-passage cells transition from OXPHOS towards glycolysis, while late-passage cells do the opposite. Additionally, late-passage cells accumulate more ROS and genetic abnormalities

F9 embryonal carcinoma stem-like cells differentiate into primitive endoderm when treated with retinoic acid (RA) and to parietal endoderm when treated with RA and dibutyryl cAMP^4^. Our studies and those of others show that this differentiation is accompanied by an increase in GATA6, SOX7, and SOX17, and the decrease of pluripotency genes including, OCT4, REX1, and NANOG^4^. We recently reported that F9 cells differentiate to a XEN-like state and this occurs regardless of their passage number^11^. However, it is surprising that the metabolic profile between the early- and late-passage populations differed dramatically. Early-passage cells transitioned from OXPHOS metabolism towards glycolysis, whereas the opposite was seen in late-passage cells. Further examination revealed that there was dysregulation in ETC enzyme stoichiometry in the differentiated late-passage cells, which resulted in the increase in mitochondrial ROS levels. Also, late-passage cells had accumulated chromosomal abnormalities when compared to early-passage cells, and whether changes in the metabolic profile preceded these chromosomal abnormalities or vice versa remains to be determined (Fig. 1)^11^.

## Significance and future directions

Our report in Cell Death Discovery is the first to shed light on the metabolic profile of XEN-like stem cells and how their passaging influences differentiation and metabolism. Mechanistically, the pluripotency potential between the early- versus late-passage populations remains unaltered despite major differences in metabolic states, karyotypes, expression of cell cycle regulators, and proliferation rates. These differences are significant and shed invaluable light as to why it is crucial to determine as many physiological parameters of a stem cell population prior to moving forward its utility as a therapeutic tool for regenerative medicine.
